# Medical adhesive-related skin injury in cancer patients: A prospective cohort study*

**DOI:** 10.1590/1518-8345.5227.3500

**Published:** 2021-11-08

**Authors:** José Ferreira Pires-Júnior, Tânia Couto Machado Chianca, Eline Lima Borges, Cissa Azevedo, Giovana Paula Rezende Simino

**Affiliations:** 1 Universidade Federal de Minas Gerais, Escola de Enfermagem, Belo Horizonte, MG, Brazil.

**Keywords:** Nursing, Intensive Care Units, Medical Oncology, Wounds and Injuries, Incidence, Patient Safety, Enfermería, Unidades de Cuidados Intensivos, Oncología Médica, Heridas y Lesiones, Incidencia, Seguridad del Paciente, Enfermagem, Unidades de Terapia Intensiva, Oncologia, Ferimentos e Lesões, Incidência, Segurança do Paciente

## Abstract

**Objective:**

to estimate the incidence of medical adhesive-related skin injury in the peripheral venous catheter fixation region in critical cancer patients, to identify risk factors, and to establish a risk prediction model for its development.

**Method:**

a prospective cohort study with a sample of 100 adult and aged patients hospitalized in an intensive care unit. The data were analyzed using descriptive, bivariate and multivariate statistics with Cox regression.

**Results:**

the incidence of medical adhesive-related skin injury was 31.0% and the incidence density was 3.4 cases *per* 100 people-days. The risk factors were as follows: alcoholism, smoking habit, hospitalization due to deep vein thrombosis, acute respiratory failure, immediate postoperative period, heart disease, dyslipidemia, use of antiarrhythmics, blood transfusion, friction injury, pressure injury, turgor, edema, hematoma, petechiae, low values in the Braden scale, clinical severity of the patient, elasticity, moisture, texture and color. The predictive model consisted in the following: decreased skin turgor, presence of hematoma and edema.

**Conclusion:**

medical adhesive-related skin injury at the peripheral venous catheter insertion site has a high incidence in critical cancer patients and is associated with decreased turgor, presence of hematoma and edema, evidence that can support the clinical practice.

## Abstract (es)

**Objetivo:**

estimar la incidencia de lesión cutánea relacionada con adhesivos médicos en la región de fijación del catéter venoso periférico en pacientes oncológicos críticos, identificar factores de riesgo y establecer un modelo de predicción de riesgo para su desarrollo.

**Método:**

cohorte prospectiva con una muestra de 100 pacientes adultos y adultos mayores ingresados en una unidad de terapia intensiva. Los datos se analizaron mediante estadística descriptiva, bivariada y multivariada con regresión de Cox.

**Resultados:**

la incidencia de lesión cutánea relacionada con adhesivos médicos fue del 31,0% y la densidad de incidencia fue de 3,4 casos por 100 personas-día. Los factores de riesgo fueron: alcoholismo, tabaquismo, hospitalización por trombosis venosa profunda, insuficiencia respiratoria aguda, postoperatorio inmediato, cardiopatía, dislipidemia, uso de antiarrítmicos, transfusión de sangre, lesión por fricción, lesión por presión, turgencia, edema, hematoma, petequias, bajos valores en la escala de Braden, gravedad clínica del paciente, elasticidad, humedad, textura y coloración. El modelo predictivo estuvo compuesto por: disminución de la turgencia cutánea, presencia de hematomas y edema.

**Conclusión:**

la lesión cutánea relacionada con adhesivos médicos en el sitio de inserción del catéter venoso periférico tiene una alta incidencia en pacientes oncológicos críticos y se asocia a disminución de la turgencia, presencia de hematoma y edema, dicha evidencia puede servir de base para la práctica clínica.

## Abstract (pt)

**Objetivo:**

estimar a incidência de lesão de pele relacionada a adesivo médico em região de fixação de cateter venoso periférico em pacientes oncológicos críticos, identificar fatores de risco e estabelecer modelo de predição de risco para o seu desenvolvimento.

**Método:**

coorte prospectiva com amostra de 100 pacientes adultos e idosos internados em unidade de terapia intensiva. Os dados foram analisados por estatística descritiva, bivariada e multivariada com regressão de Cox.

**Resultados:**

a incidência da lesão de pele relacionada a adesivo médico foi de 31,0% e a densidade de incidência foi de 3,4 casos por 100 pessoas-dia. Os fatores de risco foram: etilismo, tabagismo, internação por trombose venosa profunda, insuficiência respiratória aguda, pós-operatório imediato, cardiopatia, dislipidemia, uso de antiarrítmico, hemotransfusão, lesão por fricção, lesão por pressão, turgor, edema, hematoma, petéquias, valores baixos na escala de Braden, gravidade clínica do paciente, elasticidade, umidade, textura e coloração. Compuseram o modelo preditivo: turgor de pele diminuído, presença de hematomas e edema.

**Conclusão:**

a lesão de pele relacionada a adesivo médico em sítio de inserção de cateter venoso periférico tem elevada incidência em pacientes oncológicos críticos e está associada ao turgor diminuído, presença de hematoma e edema, evidências essas que podem embasar a prática clínica.

## Introduction

An incorrect decision regarding the type of medical adhesive and its inadequate application or removal are factors that can cause harms to the superficial layers of the skin. When the skin layers are removed along with the device, there is a skin injury related to medical adhesive, internationally known as *Medical Adhesive-Related Skin Injury* (MARSI)^(1-4)^. This injury is characterized by continuous erythema 30 minutes after removal of the medical adhesive and can be associated with the presence of vesicles, phlyctenas, erosions or ruptures in the skin^(2)^.

MARSIs affect people belonging to different age groups, from the healthiest ones in outpatient care to individuals in need of critical care^(5-8)^. It is known that injuries are often poorly recognized and, therefore, underreported, despite their high prevalence and incidence^(5-8)^, with occurrence rates of up to 29.83%^(9)^.

It is suspected that critically-ill patients, especially cancer patients, may be at greater risk for the development of MARSIs as they present alterations in their immune, circulatory and respiratory systems which cause changes in the skin structure^(10-15)^. These are patients who frequently have their skin exposed to medical adhesives, especially those used for the fixation of central and peripheral access venous catheters.

Venous infusion therapy is one of the most common health interventions in the clinical practice and it is estimated that 80% of the hospitalized patients have a Peripheral Venous Catheter (PVC). This is a device used for venous hydration, administration of blood products and medications, including chemotherapy drugs, among others^(16)^.

Scientific evidence is still scarce to confirm the dimension of the problem that MARSI represents, and which factors are related to its occurrence, to support decision-making with a view to the best practices to be adopted for its prevention. This knowledge gap can contribute to health professionals neglecting such type of injury, which is considered a preventable adverse event^(8)^.

It is understood that knowledge about MARSI, especially regarding its occurrence and risk factors, can contribute to fostering the discussion about the most common types of injury verified at the peripheral venous catheter fixation site. Proposing preventive measures can contribute to patient safety, as well as to planning and administration of the health services, consequently improving care quality in hospital institutions.

Mitigation of MARSI with assertive measures for its prevention can reduce costs and pain related to injuries, favoring qualified Nursing care and evidence-based practice.

This study aimed at estimating the incidence of medical adhesive-related skin injury in the peripheral venous catheter fixation region in critical cancer patients, to identify risk factors, and to establish a risk prediction model for its development.

## Method

### Study design

This is a prospective cohort study, carried out in an Intensive Care Unit (ICU) of a large-size cancer hospital in Belo Horizonte, Minas Gerais, Brazil, following the guidelines of the initiative called Strengthening the Reporting of Observational Studies in Epidemiology (STROBE).

### Study locus

The study was carried out in the ICU of a high-complexity philanthropic hospital specialized in cancer care with 30 beds for adult intensive care.

### Population and sample

The population consisted of all the adult and aged patients with a medical diagnosis of cancer, of both genders, hospitalized in the ICU between October 2019 and January 2020. The sample size was estimated for an infinite population, using a conservative criterion, with a confidence level of 95%, a margin of error of 5%, 20% of losses and a proportion of interest of 15.5% in the incidence of injury due to medical adhesive^(17)^. Sample calculation established a minimum number of 100 patients. Time zero (0) for data collection was defined as the day on which the PVC puncture was performed in each cancer patient hospitalized in the ICU. The follow-up time was 3 months.

The inclusion criteria were as follows: having the PVC punctured in the ICU, PVC fixed with transparent sterile polyurethane film, and remaining in the ICU after admission to the study (PVC puncture) for at least 48 hours after the first evaluation.

The total population consisted of 338 patients: 107 were using a central venous catheter, 100 were discharged or died within 48 hours after PVC puncture, 20 patients had a previous MARSI in the hand and forearm regions, and 11 did not accept to participate in the study. The non-probabilistic sample consisted of 100 patients.

### Study variables

The outcome variable was PVC-related injury due to medical adhesive in cancer patients. The independent variables were as follows: sociodemographic (age, gender and skin color); clinical (use of alcohol and tobacco, reason for hospitalization in the sector, comorbidities, medications used, neoadjuvant treatment, serum hemoglobin and albumin and blood glucose); skin characteristics (moisture, texture, turgor, edema, color, elasticity, sensitivity, presence of hair, injury due to medical adhesive, type of injury due to medical adhesive); assessment of severity and demand for care (Therapeutic Intervention Scoring System-28 - TISS-28), in addition to the risk factors (composed of the items assessed by the Braden Scale).

### Data collection instrument

The instrument that included the necessary information was elaborated by the researchers and contemplated items related to the sociodemographic and clinical variables and to the skin characteristics. It consisted of 42 items and was evaluated regarding content and appearance by two stomatherapist nurses who judged it to be adequate, clear and objective.

The Braden scale has been used to obtain the risk factors for MARSI; it has six subscales (sensory perception, activity, moisture, nutrition, friction and shear) covering intrinsic and extrinsic risk factors that, when present, indicate risk of developing pressure injury^(18)^. The Braden scale score was considered to assess medical adhesive-related skin injuries, since both conditions share similar risk factors. It is suggested that pressure injury prevention is associated with the reduction of other types of injuries that affect the skin^(1,10)^.

Thus, although each injury has its own etiology, care regarding prevention of the risk factors in common can lead to a reduction of other types of injuries^(8)^. Although the Braden scale was not specifically developed to assess the risk of MARSI, it includes items for the identification of risk factors, such as nutritional status and skin moisture.

The Therapeutic Intervention Scoring System-28 (TISS-28) was used to identify the patient›s severity and demand for care. This instrument was built to establish the Nursing workload in the ICU based on the determination of the therapeutic interventions, complexity of the Nursing care required and time spent in performing Nursing procedures in the care of critically-ill patients^(19)^.

### Data collection

Before starting data collection, the sector’s nurses were trained by the main researcher of this study (a specialist in stomatherapy). This training was based on the consensus entitled “Medical adhesives and patient safety: State of Science”^(2)^. The main topics addressed were conceptual and operational aspects in order to standardize the team in identifying the characteristics and classification of MARSI, guidance on completing the data collection instrument, photographic record of the injuries and clarification of doubts.

The information related to the sociodemographic variables, patient severity (TISS-28) and risk factors for injuries (Braden scale) was collected daily from the electronic medical records. The Braden scale and TISS-28 scores were collected on the first follow-up day for each patient. Daily inspection of the patients at the PVC insertion site was performed by the stomatherapist nurse in charge and by trained supervising nurses who recorded in the instrument the characteristics of the patients’ skin, such as moisture, texture, turgor, edema, color, elasticity, sensitivity, presence of hair, injury due to medical adhesive, and type of injury due to medical adhesive.

A specially designed protocol was used to document the assessment of the skin from the upper limbs. It contained the following variables: moisture (normal, dry, sweating, oily); texture (normal, thin, rough, wrinkled); turgor (normal/decreased); edema (yes/no); color (pink, pale, erythrosis, Raynaud’s phenomenon, jaundice, cyanosis); hematoma (yes/no); petechiae (yes/no) and hair (yes/no). All the supervising nurses from the three work shifts were trained in order to standardize the assessment.

If there was a need to change the device during the night or on weekends and holidays, the nurses were instructed to carry out a photographic record of the punctured limb after removing the device and before the new puncture for further analysis by the stomatherapist nurse and classification of the injury. The nurses also documented the evolution of the skin characteristics in the Nursing Evolution form, which enabled monitoring the patient during 24 hours a day.

### Data treatment and analysis

The data were entered in the Epi Info program, version 3.5.1, through double entry to verify their consistency. Subsequently, they were exported to the R program, version 3.6.2, for statistical analyses. In the descriptive analysis, distributions and frequencies, mean descriptive measures, median, standard deviation, minimum and maximum were adopted in the production of localized estimates.

The incidence and incidence rate of injury due to medical adhesive related to PVC were determined using the following formula: number of new cases in a given time period/number of people at risk of developing the condition in the same time period x 100. Incidence density was calculated from the number of new cases in a given time period/total number of patients-observation time.

Bivariate analysis was employed to compare the groups of patients with and without MARSI in relation to the clinical, sociodemographic and skin characteristics variables, taking into account the mean and standard deviation obtained.

The data were tested regarding normality using the Kolmogorov-Smirnov test. For the analysis of the categorical variables, Pearson’s Chi-square test or Fisher’s exact test were used. A p-value ≤ 0.05 was considered statistically significant. The association strength between the risk factors and the outcome was estimated through the Hazard Ratio (HR) measure, including 95% confidence intervals. Calculation of the significance values for each factor analyzed was also performed.

The multivariate analysis was conducted using the Cox regression model to identify the variables that exerted an influence on the development of injury due to medical adhesive in the follow-up period. Variables with a p-value ≤ 0.20 in the bivariate analysis were included in the multivariate analysis model. The procedure was repeated until all the variables present in the model had statistical significance (p ≤ 0.05), which delimited the final adjusted model.

### Ethical aspects

The study complies with Resolution No. 466/12 of the National Health Council, being approved by the Ethics and Research Committee under CAAE protocol - 15181119.0.0000.5149.

## Results

It was found that 31 patients in the sample presented a skin injury around the PVC insertion site. The global incidence of medical adhesive-related skin injury was 31.0% and the density was 3.4 cases *per* 100 people-days, varying between 1.9 and 3.7 cases *per* 100 patients-days, with a 95% confidence interval. It was verified that 57 (57%) of the patients were male. Their age was between 26 and 89 years old with a mean of 63.10 years old (± 14.98). In addition to that, 52 (52%) were brown-skinned.

It was found that 64 (64%) patients reported no consumption or being in abstinence from alcohol and 75 (75%) from smoking. All of them used medications during their hospitalization, either to control the disease that motivated the hospitalization and the associated diseases or to prevent complications. The most frequent medications that can change the skin characteristics identified were anticoagulants (92.0%) and antibiotics (78.0%).

The main reasons for hospitalization of the patients in the ICU were immediate postoperative period (35.0%) and sepsis (28.0%). The majority presented systemic arterial hypertension (74.0%) as comorbidity. Most used neoadjuvant treatment with chemotherapy drugs; and presented serum albumin (87.0%) and hemoglobin (93.0%) levels below the reference value, as well as blood glucose values (66.0%) within the normal range.

The results obtained with the application of the Braden scale were as follows: 50 (50.0%) patients with low or moderate risk and another 50 (50.0%) with high or very high risk. The scores obtained by applying TISS-28 were as follows: 60 (60.0%) with score I or II and 40 (40.0%) with score III or IV.

The mean time until occurrence of MARSI found was 5.60 (± 5.49) days, with a minimum of 2 days and a maximum of 8 days. In 21 (68.0%) patients, the medical adhesive-related skin injury was classified as skin removal. As for the location of the medical adhesive-related skin injuries, 100% were in the upper limbs.

The main skin characteristics in the PVC region observed in most of the patients were absence of edema (55.0%), hematoma (78.0%), petechiae (81.0%) and hair (86.0%), as well as pink color (61.0%).

The variables that presented a statistically significant association (p ≤ 0.05) with the presence of injury due to medical adhesive were alcoholism, smoking habit, diagnosis of deep vein thrombosis and acute respiratory failure at admission, being in the immediate postoperative period, presence of heart disease, dyslipidemia, use of antiarrhythmics, having received packed red blood cells, presence of friction injury, pressure injury, decreased turgor, edema, hematoma, petechiae, risk scores in the Braden scale, clinical severity of the patient, elasticity, moisture, texture and color (Table 1).

**Table 1:** Variables with a significant association with the outcome of skin injury due to medical adhesive related to peripheral venous catheter. Belo Horizonte, MG, Brazil, 2020

Variables	Injury due to medical adhesive						p-value	**HR** * **[95% CI]** ^†^
	Yes		No		Total			
	N	%	N	%	N	%		
**Alcoholism**								
No/Abstinence	21	68.00%	43	62.30%	64	64.00%	**0.011**	0.38 (0.14 - 1.05)
Yes	10	32.30%	26	37.70%	36	36.00%		
**Smoking habit**								
No/Abstinence	22	71.00%	53	76.80%	75	75.00%	**0.044**	3.75 (1.27 - 11.2)
Yes	9	29.00%	16	23.20%	25	25.00%		
**Deep venous thrombosis**								
No	26	83.90%	67	97.10%	93	93.00%	**0.016**	6,442 (1,175 - 35,308)
Yes	5	16.10%	2	2.90%	7	7.00%		
**Acute respiratory failure**								
No	25	80.60%	66	95.70%	91	91.00%	**0.015**	5,280 (1,226 - 22,743)
Yes	6	19.40%	3	4.30%	9	9.00%		
**Surgical IPOP** ^‡^								
No	26	83.90%	36	52.20%	62	62.00%	**0.003**	0.209 (0.072 - 0.610)
Yes	5	16.10%	33	47.80%	38	38.00%		
**Heart disease**								
No	27	87.10%	47	68.10%	74	74.00%	**0.045**	0.316 (0.098 - 1.015)
Yes	4	12.90%	22	31.90%	26	26.00%		
**Dyslipidemia**								
No	29	93.50%	48	69.60%	77	77.00%	**0.008**	0.157 (0.034 - 0.722)
Yes	2	6.50%	21	30.40%	23	23.00%		
**Antiarrhythmic**								
No	28	90.30%	48	69.60%	76	76.00%	**0.025**	0.245 (0.067 - 0.895)
Yes	3	9.70%	21	30.40%	24	24.00%		
**Packed red blood cells**								
No	14	45.20%	46	66.70%	60	60.00%	**0.042**	2,428 (1,021 - 5,776)
Yes	17	54.80%	23	33.30%	40	40.00%		
**Braden**								
Very high/High	22	70.90%	28	40.60%	50	50.00%	**0.008**	1.06 (1.03 - 1.08)
Moderate/Low	9	29.10%	41	59.40%	50	50.00%		
**TISS-28** ^§^								
I/II	7	22.60%	53	76.81%	60	60.00%	**<0.001**	1.70 (1.40 - 1.65)
III/IV	24	77.40%	16	23.19%	40	40.00%		
**Friction injury**								
No	25	80.60%	66	95.70%	91	91.00%	**0.015**	5,280 (1,226 - 22,744)
Yes	6	19.40%	3	4.30%	9	9.00%		
**Pressure injury**								
No	11	35.50%	60	87.00%	71	71.00%	**<0.001**	12,121 (4,388 - 33,479)
Yes	20	64.50%	9	13.00%	29	29.00%		
**Dermatitis associated with incontinence**								
No	10	32.30%	69	100%	79	79.00%	**<0.001**	284,619 (16,008 - 5,060.271)
Yes	21	67.70%	0	0.00%	21	21.00%		
**Turgor**								
Diminished	30	96.80%	9	13.00%	39	39.00%	**<0.001**	0.005 (0.006 - 0.459)
Normal	1	3.20%	60	87.00%	61	61.00%		
**Edema**								
No	4	12.90%	51	73.90%	55	55.00%	**<0.001**	0.016 (0.003 - 0.080)
Yes	27	87.10%	18	26.10%	45	45.00%		
**Hematoma**								
No	11	35.50%	67	97.10%	78	78.00%	**<0.001**	0.006 (0.019 - 0.224)
Yes	20	64.50%	2	2.90%	22	22.00%		
**Petechiae**								
No	16	51.60%	65	94.20%	81	81.00%	**<0.001**	0.065 (0.192 - 0.224)
Yes	15	48.40%	4	5.80%	19	19.00%		
**Moisture**								
Normal	0	0.00%	46	66.70%	46	46.00%	**<0.001**	Ref. 0.006 (0.001 - 0.202)
Altered	31	100.00%	23	33.30%	54	54.00%		
**Texture**								
Normal	0	0.00%	48	69.60%	48	48.00%	**<0.001**	1.018 (0.232 - 4.465)
Altered	31	100.00%	21	30.40%	52	52.00%		
**Color**								
Normal	3	9.70%	58	84.10%	61	61.00%		1.188 (0.044 - 31.574)
Altered	28	90.30%	11	15.90%	39	39.00%	**<0.001**	

* HR = Hazard Ratio;

† CI = Confidence Interval;

‡ IPOP = Immediate Postoperative Period;

§ TISS-28 = Therapeutic Intervention Scoring System

Regarding the multivariate analysis, the variables that exerted a statistically significant and joint impact on the outcome of injury due to medical adhesive and that comprised the final predictive model for the presence of injury due to medical adhesive in the patients hospitalized in the Oncology ICU under study during the period were decreased turgor, presence of hematoma and edema (Table 2).

**Table 2:** Risk factors associated with the time until occurrence of injury due to medical adhesive. Belo Horizonte, MG, Brazil, 2020

Independent variables	**HR** *	**95% CI** ^†^ **HR** *		p-value
		Lower	Upper	
Decreased turgor	0.53	0.25	0.86	0.024
Hematoma	0.61	0.32	1.86	0.004
Edema	0.71	0.63	3.09	0.002

* HR = Hazard ratio;

† CI = Confidence Interval

## Discussion

This study provided new knowledge about the integrity of altered skin related to medical adhesive at the PVC insertion site in critical cancer patients, since MARSI incidence studies prevail in neonate patients^(20)^, older adults^(17)^, during the postoperative period^(7)^ and with stomas^(21)^.

The incidence of medical adhesive-related skin injuries found in this study is similar to that identified in older adults hospitalized in a long-term care institution in Japan (cumulative incidence of 15 ± 5% and incidence density of 38/1,000 people-days)^(17)^ and higher than that obtained in a vascular wound clinic in the United States (incidence of 5.8%)^(7)^. However, due to the scarce number of publications on the theme, whether due to differences in the context of the population evaluated and scenarios analyzed, types of medical adhesives used, or to differences in the methods used and in the variability of the results found, the comparison of the MARSI incidence values has been hampered^(5,7,9,17)^. In addition to that, another problem is the assessment of MARSI since, in adult inpatient units, it has been observed that the majority of MARSI-type skin injuries were wrongly classified as skin denudation (48%)^(8)^ and in upper limbs^(8-9)^.

In this sense, this research represents new knowledge about the integrity of altered skin related to a medical adhesive in the PVC insertion site, as it involves cancer patients in an intensive care setting.

It is verified that MARSI occurs in people of different ages, with diverse clinical problems and assisted at all health care levels^(7,17,20)^, it can lead to disastrous consequences on the patients’ physical conditions and negatively impact on safety and satisfaction with the services offered, in addition to causing socioeconomic impacts on the health services and society^(8)^.

It is observed that critically-ill patients are at greater risk of alterations in coagulation, tissue perfusion, inflammation, immune function, metabolism, nutrition and use of drug therapies that lead to changes in their biological responses and influence skin healing^(21)^. Regarding the critical cancer patients, multiple drug therapies have deleterious effects on them^(1-8)^ and the combination of chemotherapy with the specificities of the cancer disease makes the skin of critical cancer patients even more fragile, making them more susceptible to MARSIs^(9)^, which was proved in this study.

What has already been stated in the literature has been verified, proving that, in this population, changes in the epidermis, dermis and connective tissue can be associated with the use of antineoplastic agents and with concomitant factors such as age, smoking habit, chronic diseases and antineoplastic treatment, which can lead to changes in the healing process^(11-12)^. Also relevant is the evaluation of the time until the occurrence of MARSI. It was found that the risk factors of edema, hematoma and decreased turgor presented a statistically significant association (p < 0.05) with time until occurrence of MARSI and predisposed to a greater risk for its development (HR ≥ 1) in critical cancer patients.

The presence of edema in these patients can be associated with several clinical conditions such as allergic responses, congestive heart failure, deep vein thrombosis, hypoalbuminemia, use of chemotherapy agents, corticotherapy, sepsis and fluid overload. Thus, patients with greater clinical severity presented the outcome of Medical Adhesive-Related Skin Injury (MARSI)^(4,7)^.

Edema was a predictive factor for MARSI. It is noteworthy that, allied to the skin distension caused by the edema, the memory effect associated with some medical adhesives contributes to the occurrence of skin injuries associated with medical adhesives, such as blisters^(4,7)^. Edema is found to weaken the junction between the dermis and epidermis, increasing the risk of injury during removal of the medical adhesive^(8)^.

In this study, it was also identified that hematoma was a predictive factor for developing MARSI. This is usually caused by the rupture of blood vessels that cause small hemorrhages under the skin and lead to a reduction in the supply of oxygen and nutrients, which increases skin fragility and can favor the risk of injuries^(4,21-22)^. In the literature, the presence of hematoma was not identified as a risk factor for the development of MARSI, a fact that was refuted in this study.

Another predictive factor for MARSI found in this study was decreased turgor. This is indicative of dehydrated and dry skin due to reduced production of sebaceous and sweat glands, which increases the risk of impaired skin integrity^(22-23)^. Turgor is particularly important in skin assessment, since specific Nursing interventions for the prevention of MARSI, according to the skin type, must be prescribed and implemented^(3)^.

In clinical health care settings, some medical devices such as the PVC are routinely used and require repeated applications and removal of medical adhesives for their fixation on the patients’ skin. In this sense, the risk of impaired skin integrity is increased, especially in the case of critical cancer patients^(11-12)^. The association between skin antisepsis and adhesive selection and fixation increases the risk of injuries due to medical adhesives. We know that antimicrobial skin preparation is valuable in the antisepsis process; however, it modifies the microbiota and, consequently, the pH of the skin, in addition to a mechanical friction process in the use of these preparations under the skin, which causes friction on its surface^(4,9)^. The repercussion of this effect on the potentiation of the harms to the skin is not yet fully understood, with lack of studies for its measurement.

It is verified that the predictive factors for medical adhesive-related skin injury in the PVC fixation region found in this study are considered as potential for changing adhesion between the skin and the medical adhesive, making it stronger than the fixation of skin cells to the skin. Cohesive failure occurs when the adhesive strength exceeds that of the interactions between the several skin layers. As a result, the epidermal layers get separated or the epidermis completely separates from the dermis^(4)^.

Despite the relevance of extrinsic factors related to MARSI, such as the type of adhesive and method of removal and fixation of such input, it is perceived that individual intrinsic characteristics need to be considered, as they interfere with tissue structuring and with the dermal-epidermal junction.

The following were identified as limitations of this study: the fact that the techniques for applying and removing the medical adhesive were not evaluated, in addition to the analysis of the use of the “Protocol of care measures with peripheral venous access” in the clinical practice. It is recommended that tests to evaluate allergy to the adhesives to be standardized in the institutions be routinely employed for the patients, since allergic reactions are common.

In addition to that, given that the study setting was an ICU of a large-size cancer hospital and that this may not represent the profile of the patients in other care settings, subsequent research studies are suggested in order to establish incidence values and risk factors for MARSI and to enable comparative studies in different care settings and with different populations.

MARSI is an adverse event that has major implications for patient safety and for the quality of Nursing care provided, with ethical and legal implications for both professionals and institutions. In this sense, the study collaborates with the Nursing practice in the care of cancer patients hospitalized in ICUs and who may suffer from the possibility of developing these injuries. Thus, it shows the importance of careful skin evaluation, especially for patients with PVC protected by medical adhesives, since these can lead to skin injuries.

## Conclusion

In this study, an incidence of medical adhesive-related skin injury at the PVC insertion site of 31.0% was found, as well as an incidence density of 3.4 cases *per* 100 people-days. The predictive model in the multivariate analysis consisted of the decreased turgor, presence of bruises and edema variables.

Medical adhesive-related skin injury at the PVC insertion site has a high incidence in critical cancer patients and is associated with decreased turgor, presence of hematoma and edema in these individuals.

Thus, the study is valuable insofar as the clinical evidence generated can favor the clinical practice in the assistance provided to patients at risk of impaired skin integrity.

## References

[1] Tiggelen HV, Damme NV, Theys S, Vanheyste E, Verhaeghe S, LeBlanc K (2020). The prevalence and associated factors of skin tears in Belgian
nursing homes: A cross-sectional observational study. J Tissue Viability.

[2] McNichol L, Lund C, Rosen T, Gray M (2013). Medical Adhesives and Patient Safety. Orthop Nurs.

[3] Hitchcock J, Savine L (2017). Medical adhesive-related skin injuries associated with vascular
access. Br J Nurs.

[4] Fumarola S, Allaway R, Callaghan R, Collier M, Downie F, Geraghty J (2020). Overlooked and underestimated: medical adhesive-related skin
injuries. Best practice consensus document on prevention. J Wound Care.

[5] Kelly-O’Flynn S, Mohamud L, Copson S (2020). Medical adhesive-related skin injury. Br J Nurs.

[6] Li Q, Wang H, Liu F, He Y (2020). Investigation and Analysis of Catheter-Associated Skin Impairment
in PICC’s Patients in Jingzhou City. J Biosci Med.

[7] Ratliff CR (2017). Descriptive study of the frequency of medical adhesive-related
skin injuries in a vascular clinic. J Vasc Nurs.

[8] Wang D, Xu H, Chen S, Lou X, Tan J, Xu Y (2019). Medical Adhesive-Related Skin Injuries and Associated Risk
Factors in a Pediatric Intensive Care Unit. Adv Skin Wound Care.

[9] Zhao H, Yu H, Huang H, Ling Y, Zhou X, Wei Q (2018). Prevalence of medical adhesive-related skin injury at
peripherally inserted central catheter insertion site in oncology
patients. J Vasc Access.

[10] Vasconcelos JMB, Caliri MHL (2017). Ações de enfermagem antes e após um protocolo de prevenção de
lesões por pressão em terapia intensiva. Esc Anna Nery.

[11] Pradelli J, Verdoire P, Boutros J, Frin AC, Follana P, Duquesne J (2020). Allergy evaluation of hypersensitivity to platinium salts and
taxanes: a six-year experience. J Allergy Clin Immunol Pract.

[12] Pagani M, Bavbek S, Dursun AB, Bonadonna P, Caralli M, Cernadas J (2019). Role of Skin Tests in the Diagnosis of Immediate Hypersensitivity
Reactions to Taxanes: Results of a Multicenter Study. J Allergy Clin Immunol Pract.

[13] Morgan R, Parsad S, Turaga KK, Eng OS (2020). HIPEC with Cisplatin in a Patient with a Prior Hypersensitivity
Reaction to Systemic Oxaliplatin. Basic Clin Pharmacol Toxicol.

[14] Ferreira DH, Teixeira MLO, Branco EMSC (2017). Nursing care in the prevention of adhesive-related skin injuries
in surgical wounds. Ciênc Cuidado Saúde.

[15] National Pressure Injury Advisory Panel (NPIAP) (2020). About the NPIAP. [Internet].

[16] Webster J, Osborne S, Rickard CM, Marsh N (2019). Clinically-indicated replacement versus routine replacement of
peripheral venous catheters. Cochrane Database Syst Rev.

[17] Konya C, Sanada H, Sugama J, Okuwa M, Kamatani Y, Nakagami G (2010). Skin injuries caused by medical adhesive tape in older people and
associated factors.

[18] Paranhos WY, Santos VLCG (1999). Risk assessment for pressure ulcers using the Braden Scale in
Portuguese. Rev Esc Enferm USP.

[19] Velozo KDS, Garcia PCR, Piva JP, Fiori HH, Cabral DD, Einloft PP (2017). Scores TISS-28 versus NEMS to size the nursing team in a
pediatric intensive care unit. Einstein (São Paulo).

[20] Kim MJ, Jang JM, Kim HK, Heo HJ, Jeong IS (2019). Medical Adhesives-Related Skin Injury in a Pediatric Intensive
Care Unit. J Wound Ostomy Continence Nurs.

[21] LeBlanc K, Whiteley I, McNichol L, Salvadalena G, Gray M (2019). Peristomal Medical Adhesive-Related Skin Injury: Results of an
International Consensus Meeting. J Wound, Ostomy Continence Nurs.

[22] Rayner RL, Carville KJ, Leslie GD, Dhaliwal SS (2019). Clinical purpura and elastosis and their correlation with skin
tears in an aged population. Arch Dermatol Res.

[23] Silva CVB, Campanili TCGF, Freitas NO, LeBlanc K, Baranoski S, Santos VLCG (2020). ISTAP classification for skin tears: Validation for Brazilian
Portuguese. Int Wound J.

